# MRgHIFU treatment in pain palliation of bone metastases: initial experience from the Rizzoli Orthopaedic Institute

**DOI:** 10.1186/2050-5736-2-S1-A15

**Published:** 2014-12-10

**Authors:** Alberto Bazzocchi, Alessandro Napoli, Paolo Spinnato, Giancarlo Facchini, Danila Diano, Maurizio Busacca, Carlo Catalano, Ugo Albisinni

**Affiliations:** 1 The "Rizzoli" Institute, Bologna, Italy; 2 University of Bologna, Italy; 3 University of Rome "La Sapienza, Italy

## Background

The increasing longevity of the population coupled with the progress of treatments for primary tumors is enhancing the incidence and the burden of distant metastases such as bone metastases. Bone metastases are the most common cause of cancer-related pain. Quality of life may be significantly impaired as a consequence of painful bone metastases. The treatment of secondary bone lesions will become even more important in the near future. The development of strategies to improve quality of life in patients with bone metastases is fundamental and represents a major clinical challenge. This issue is of huge impact on today health care and economy.

## Purpose of the study

The aim of this work was to assess the efficacy of MRgHIFU in the treatment of pain caused by bone metastases, in the preliminary cohort of patient treated at our Institute.

## Materials and methods

From November 2012, 11 patients affected by bone metastases were enrolled in the study. Pain was scored before and after MRgHIFU (ExAblate 2100, InSightec, Israel on 1.5 T Signa Twin Speed MR system, GE, USA) using visual analogue scale (VAS, 0-10 points). The initial follow-up included clinical assessment at 1 and 3 months.

## Results

Fourteen lesions were treated in 11 patients (6 males, 5 females - age 51±7, range 47-68 years old) with different primary cancers (4 kidney, 3 breast, 2 lung, 1 thyroid, 1 colon). The treated lesions were located as follows: 11 at pelvis, 2 at femur, 1 at rib. In four patients the bone lesion was single. Treatments were performed under spinal anesthesia in 9/11 patients, under general anesthesia in 2/11. One patient died two months after the treatment for causes not related to the procedure, while five patients reached the 3-month check point. After 1 month, the average pain drop amounted to 56% (p<0.01), and after 3 months to 69% (p<0.01) with 3 out of 5 patients presenting VAS=0. The only experienced complication was prostate inflammation with urinary retention after the treatment of a pubic lesion.

## Conclusion

Our preliminary experience confirms the efficacy of MRgFUS in controlling pain of bone metastases.

